# A Rare Variant of Penile Squamous Cell Carcinoma in a Man with Paraplegia

**DOI:** 10.7759/cureus.3244

**Published:** 2018-09-03

**Authors:** Daniel C Morse, Jaime A Tschen, Sirunya Silapunt

**Affiliations:** 1 Mcgovern Medical School, University of Texas Mcgovern Medical School at Houston, Houston, USA; 2 Dermatopathology, St Joseph Dermatopathology, Houston, USA; 3 Dermatology, University of Texas Mcgovern Medical School at Houston, Houston, USA

**Keywords:** penile cancer, verrucous, squamous cell carcinoma, paraplegia, verrucous carcinoma

## Abstract

Verrucous carcinoma (VC) is a rare variant of squamous cell carcinoma (SCC). It is described as a low grade, slow growing, locally infiltrative neoplasm that accounts for 3%-8% of penile SCCs. Here we report a case of destructive VC of the glans penis in a paraplegic man resulting in a hypospadias from the tip of the glans to the corona. Histology demonstrated exophytic squamous epithelial proliferation with characteristic round, pushing borders. In situ hybridization was positive for both low-risk and high-risk strains of human papillomavirus.

## Introduction

Cancers of the penis are responsible for 0.5% of male cancers in the USA and Europe, and are nearly always a form of squamous cell carcinoma (SCC) [1-3]. Verrucous carcinoma (VC), a rare variant of SCC, was first reported in 1948 and it has been observed in the oral cavity, larynx, penis, vulva, scrotum, anus, and various cutaneous locations [4-5]. Penile verrucous carcinoma (PVC), accounts for 3%-8% of penile SCCs [6-8], and is uncommonly associated with human papillomavirus (HPV).

## Case presentation

**Figure 1:**
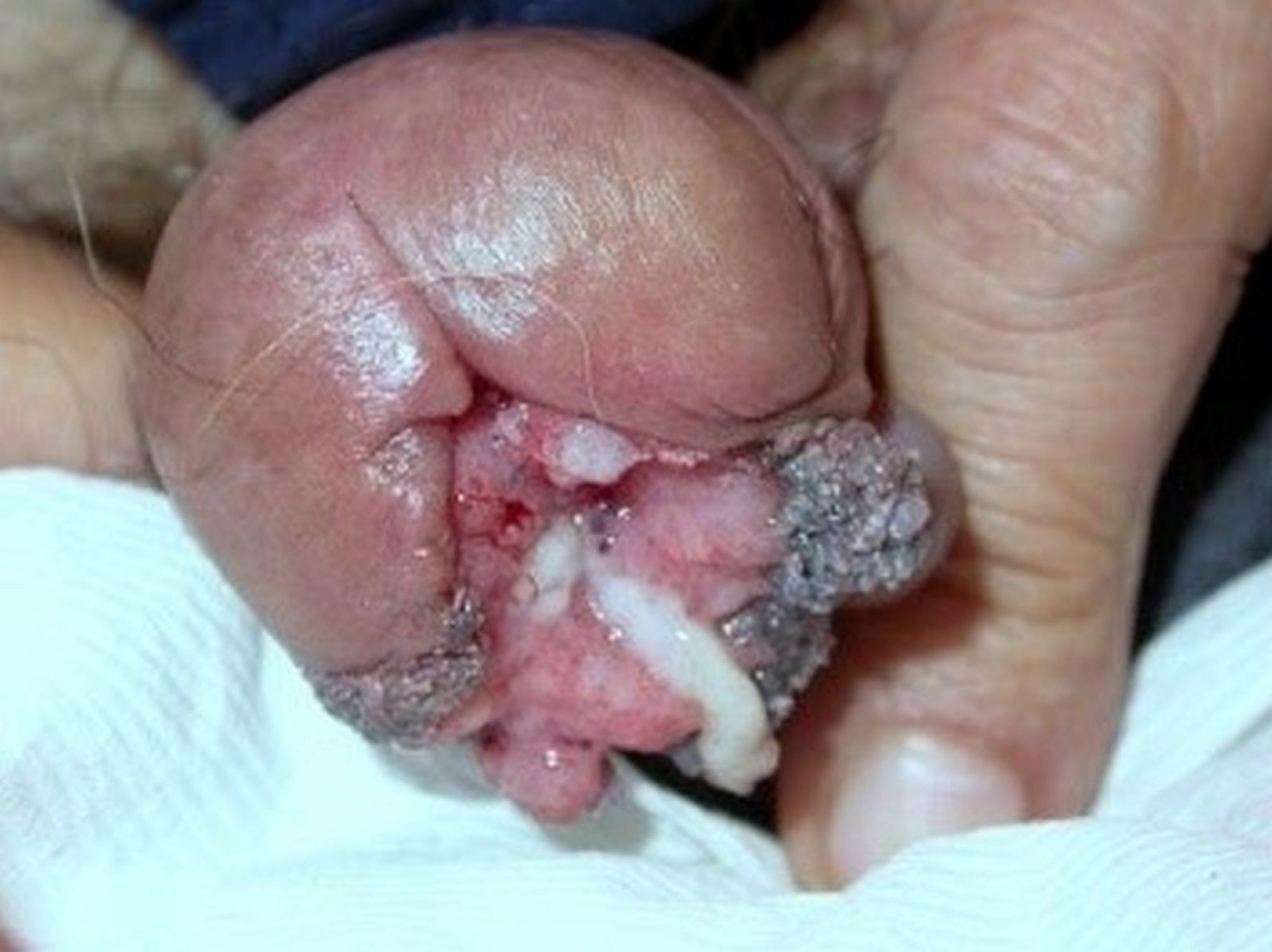
A 2.5-cm exophytic cauliflower-like penile nodule occupying the meatal orifice and ventral glans.

A 64-year-old uncircumcised, paraplegic male presented with a two-month history of an asymptomatic, enlarging penile lesion with episodic spontaneous bleeding. The patient had been confined to a wheelchair for the past 15 years due to transverse myelitis and was wearing a diaper secondary to overflow urinary incontinence. He reported a monogamous relationship with his wife and currently was not sexually active because of erectile dysfunction. The patient denied a history of smoking and sexually transmitted diseases. Past surgical history was significant for a transurethral resection of the prostate for benign prostatic hyperplasia. Physical exam revealed a 2.5-cm cauliflower-like nodule occupying 40% of the glans penis (Figure 1). The lesion expressed a white discharge and was nontender. It involved the distal urethrae creating a hypospadias from the tip of the glans to the corona. The inguinal lymph nodes were not palpable.

**Figure 2:**
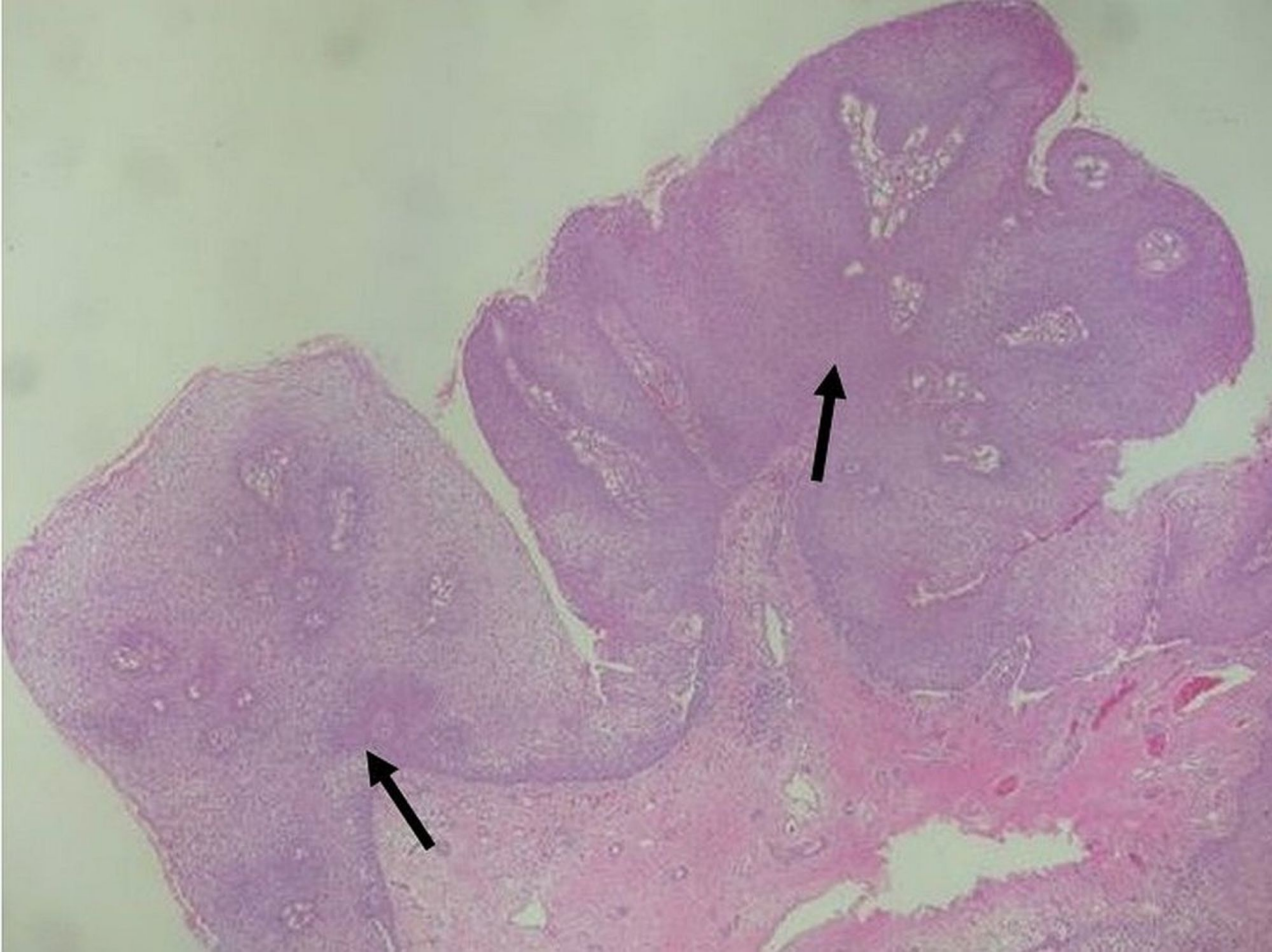
Low-power view of verrucous tumor with exophytic papillary architecture and a markedly irregular epidermal acanthosis (H&E, 10x).

**Figure 3:**
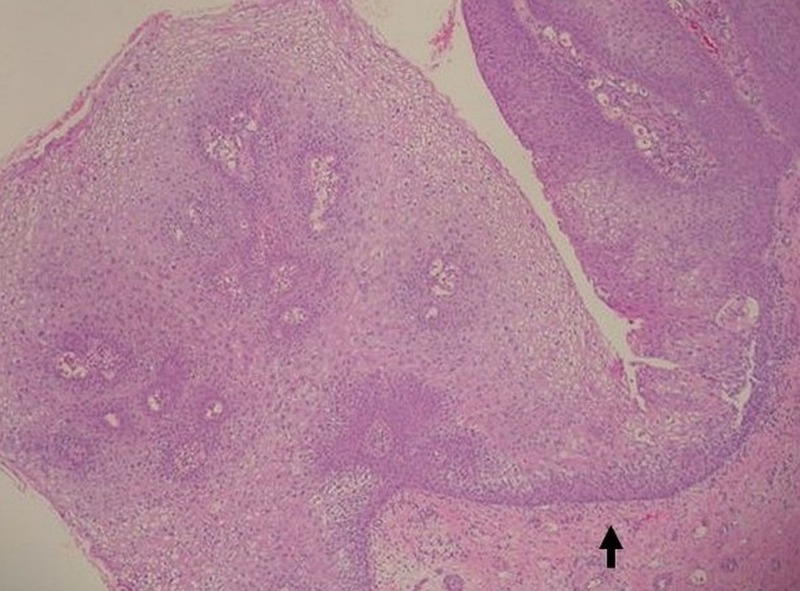
Squamous epithelial proliferation with the characteristic blunt pushing deep margins (H&E, 20x).

**Figure 4:**
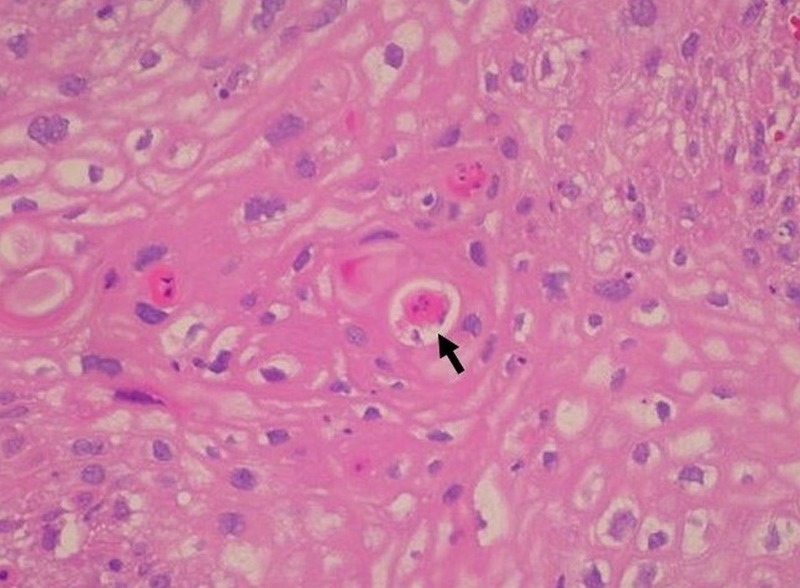
Higher power magnification demonstrates nuclear polymorphism, mild cytologic atypia, and dyskeratosis with keratin pearls (H&E, 400x).

**Figure 5:**
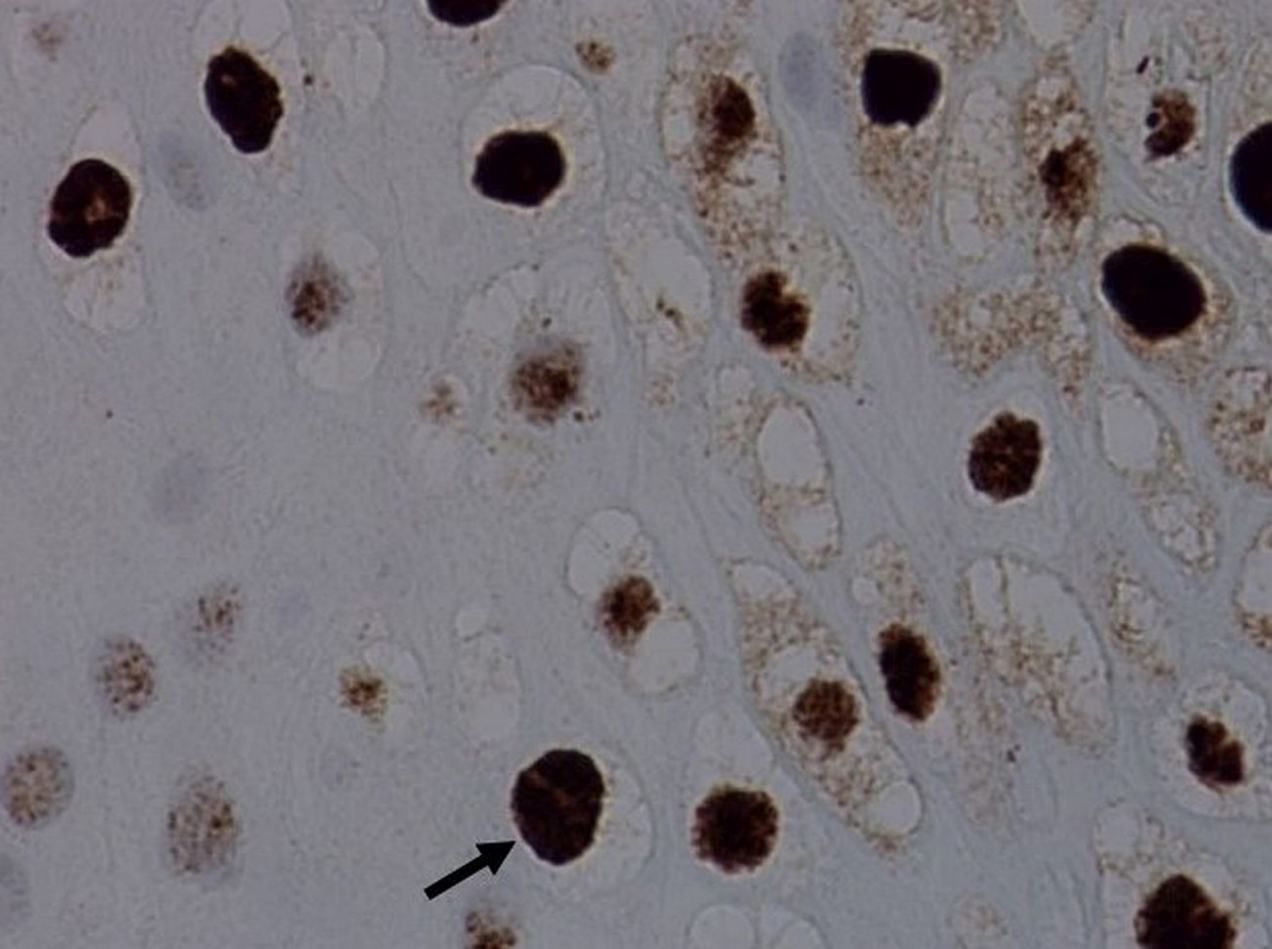
In situ hybridization of the lesion revealed strongly positive labeling of the nuclei with human papilloma virus (HPV) types 6 and 11.

**Figure 6:**
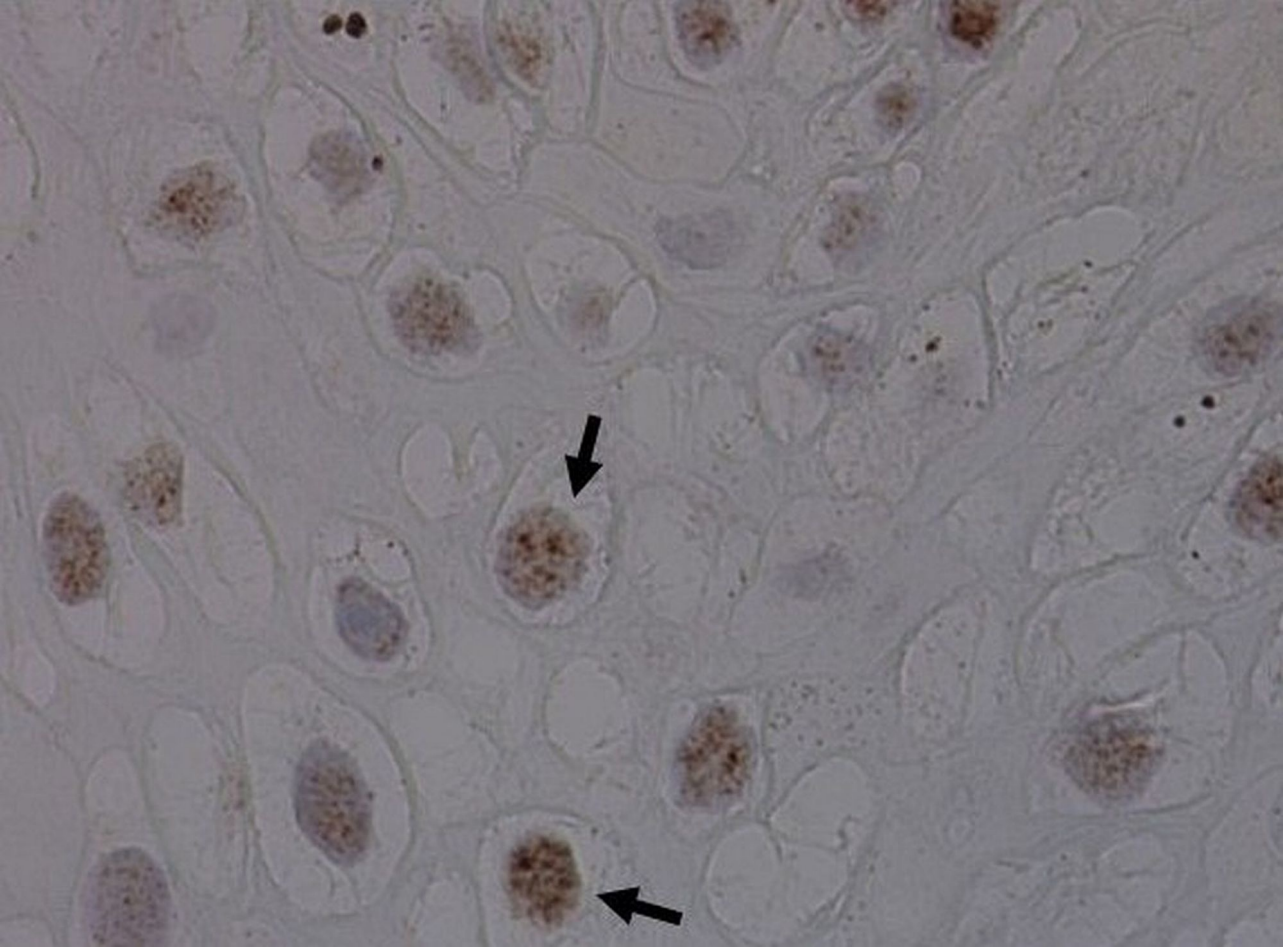
Positive labeling of the nuclei with human papilloma virus (HPV) strains 31, 33 (in situ hybridization).

Skin biopsy was performed, and histopathology demonstrated exophytic papillary architecture with markedly irregular epidermal acanthosis (Figure 2). Well-differentiated, bulbous projections of squamous cell proliferation with characteristic round, pushing deep margins were found pressing against the dermis and submucosa (Figure 3). High-power magnification revealed nuclear polymorphism, dyskeratosis with keratin pearls, and mild cytologic atypia (Figure 4). No dermal or submucosal invasion was seen. These histological features were consistent with VC. In situ hybridization was positive for HPV 6, 11, 31, and 33 and negative for HPV 16 and 18 (Figures 5-6). Venereal disease research laboratory (VDRL) and human immunodeficiency virus (HIV) screening were negative. The patient underwent a partial penectomy. Follow-up cystourethroscopy 10 months later showed no evidence of recurrence.

## Discussion

The 2016 World Health Organization guidelines suggest that penile carcinoma should be classified according to both its morphology and relation to HPV [1]. With these guidelines in mind, the differential diagnosis for our patient’s lesion included VC, giant condyloma, warty carcinoma (warty SCC), carcinoma cuniculatum and papillary carcinoma, not otherwise specified (NOS).

Chaux and Cubilla provided a detailed discussion of these various penile carcinomas [7]. Giant condyloma is marked by arborizing, nonundulating papillae, a prominent fibrovascular core, a regular, broad and pushing tumor base, superficial koilocytic atypia, and a strong association with HPV. Warty carcinoma (warty SCC) is distinguished by long and undulating, condylomatous papillae, a prominent fibrovascular core, a rounded or irregular tumor base, diffuse koilocytic atypia, and a strong association with HPV. Carcinoma cuniculatum is differentiated by straight papillae, infrequent fibrovascular cores, regular, broad and pushing tumor base, no koilocytic atypia, and no strong association with HPV. Papillary carcinoma, NOS features variably complex papillae, variably present fibrovascular cores, an irregular tumor base, no koilocytic atypia, and no strong association with HPV. Finally, VC is marked by straight papillae, infrequent fibrovascular cores, regular, broad and pushing tumor base, infrequent koilocytic atypia, and no strong association with HPV.

Penile verrucous carcinoma typically presents as an asymptomatic, slow growing, exophytic, flesh-colored, verrucous or cauliflower-like lesion most frequently on the glans of the penis [6, 9]. A large PVC can become painful due to the resulting necrosis and infection. PVC is easily misdiagnosed as verruca due to its warty appearance and thus often improperly treated with conservative therapy [4]. Distant metastases are infrequently reported, but the malignancy is locally aggressive with potential for significant local tissue destruction if left unchecked. Our patient had hypospadias from the tip of the glans to the corona as a result of tumor invasion and destruction of the urethra.

Histological examination classically reveals well-differentiated neoplastic cells, hyperkeratosis, distinctly irregular acanthosis, and papillomatosis [4, 6]. The tumor base is broad, with characteristic blunt pushing margins comprising squamous epithelial proliferations pushing against the surrounding stroma [4, 6], as was seen in our case. Due to its exophytic papillary architecture it is crucial to achieve an adequate depth when performing a biopsy, as superficial biopsies are often indistinguishable from pseudoepitheliomatous hyperplasia secondary to varying pathology [4, 8].

Poor hygiene, infection, phimosis, tight prepuce, and lack of circumcision are linked to the etiology of all penile cancers including VC [4]. The presence of these risk factors over many years leads to the typical presentation of PVC in the elderly. Our patient’s lack of circumcision combined with his daily use of diapers, due to his urinary incontinence and paraplegia, likely contributed to poor penile hygiene and increased risk for malignancy.

In contrast to the majority of SCC of the penis, PVC is infrequently associated with HPV infection [1-2, 4, 6]. HPV was found to be associated with PVC in 33.3% of 12 cases [10]. A later study by Stankiewicz et al. and a systemic review by Backes et al. reported HPV in 23% of 13 cases and 22.4% of 58 cases of PVC, respectively [6, 11]. Rubin et al. described four HPV-associated PVCs that were associated with one type of HPV, either high-risk or low-risk strains [10]. Stankiewicz et al. reported a single neoplasm with both low-risk and high-risk HPV expression. Our case characterizes an additional report of a PVC associated with both low-risk HPV types 6 and 11 as well as high-risk HPV types 31 and 33.

Treatment typically involves local excision and partial penectomy [9], while alternative treatments include glansectomy and Mohs micrographic surgery [3, 12]. As PVC rarely metastasizes, inguinal lymphadenectomy is generally not performed and prophylactic inguinal lymphadenectomy in patients with PVC is not recommended [8-9]. Interestingly, it has been reported that around 30% of VC contain micro-lesions of invasive SCC [13]. Thus, it is imperative that patients who have been treated with local excision be carefully monitored for recurrence.

## Conclusions

We report a PVC positive for both low-risk and high-risk HPV. This is significant as HPV is rarely described in PVC. Our case also highlights notable risk factors for poor penile hygiene: lack of circumcision, immobility due to paraplegia, and a history of regular diaper wearing due to urinary incontinence. While lack of circumcision has been regularly reported as a risk factor for genital malignancies, our report brings attention to the potentially increased risk of genital malignancies in patients with a history of immobility and regular diaper wearing. We recommend increased vigilance for genital malignancies in elderly patients with a history of immobility or regular diaper wearing.
